# Thoracic endovascular aortic repair (TEVAR) in a combined penetrating thoracic aortic and spinal cord injury

**DOI:** 10.1093/jscr/rjae771

**Published:** 2024-12-21

**Authors:** Jacqueline Amm, Estelle Laney, Rubinette Robbertze, Maeyane Moeng

**Affiliations:** Department of Surgery, University of the Witwatersrand, Floor 9, Charlotte Maxeke Johannesburg Academic Hospital, 17 Jubilee Rd, Parktown, Johannesburg, South Africa; Department of Trauma Surgery, University of the Witwatersrand, Floor 7, Charlotte Maxeke Johannesburg Academic Hospital, 17 Jubilee Rd, Parktown, Johannesburg, South Africa; Department of Radiology, University of the Witwatersrand, Floor 5, Charlotte Maxeke Johannesburg Academic Hospital, 17 Jubilee Rd, Parktown, Johannesburg, South Africa; Department of Trauma Surgery, University of the Witwatersrand, Floor 7, Charlotte Maxeke Johannesburg Academic Hospital, 17 Jubilee Rd, Parktown, Johannesburg, South Africa

**Keywords:** case report, thoracic aortic injury, penetrating injury, thoracic endovascular aortic repair, aortic pseudoaneurysm, Brown–Sequard syndrome

## Abstract

Endovascular repair of aortic injuries secondary to blunt trauma has been widely described. However, literature on endovascular management in penetrating aortic injuries is scarce. The patient in this case report, a victim of penetrating thoracic aortic trauma, presented 5 days after injury with Brown–Sequard syndrome and a contained aortic injury (pseudoaneurysm) and was haemodynamically stable. Therefore, thoracic endovascular aortic repair was an option in this case. Endovascular repair carries a lower peri-operative morbidity and mortality rate than open repair. However, because most cases of penetrating thoracic vascular injury have haemodynamic instability, open surgery is considered the standard of care. This case demonstrates successful management of an aortic injury with a minimally invasive procedure.

## Introduction

Thoracic aortic injuries are associated with a high mortality rate (85% regardless of the mechanism of injury) [1]. Surgical repair of the aorta can be done by primary repair of the defect, or by resecting the injured portion and vessel grafting [1]. Primary repair is performed by either an open or endovascular method [2]. Endovascular repair of aortic injuries secondary to blunt trauma has been widely described. However, literature on endovascular management in penetrating aortic injuries is scarce [2].

## Case report

Ambulance services brought in a 36-year-old man from a referral clinic, after sustaining a stab wound to the right side of his back. He was allegedly attacked and robbed while on his way to work. He presented to the clinic five days after the injury, complaining of difficulty walking due to weakness and numbness of the right leg.

On arrival at Charlotte Maxeke Johannesburg Academic Hospital, the patient was assessed according to ATLS® principles. He maintained his airway, with good bilateral air entry on auscultation, and oxygen saturation was 95% on room air. His pulse rate was 60 beats per minute, and blood pressure was 195/113 mmHg, with a Glasgow Coma Scale of 15/15. On further examination, a single incised wound was found over the right para-spinal region, at T4 level. The wound was 2 cm long; with interrupted sutures *in situ* (the wound had been sutured at the clinic).

**Figure 1:**
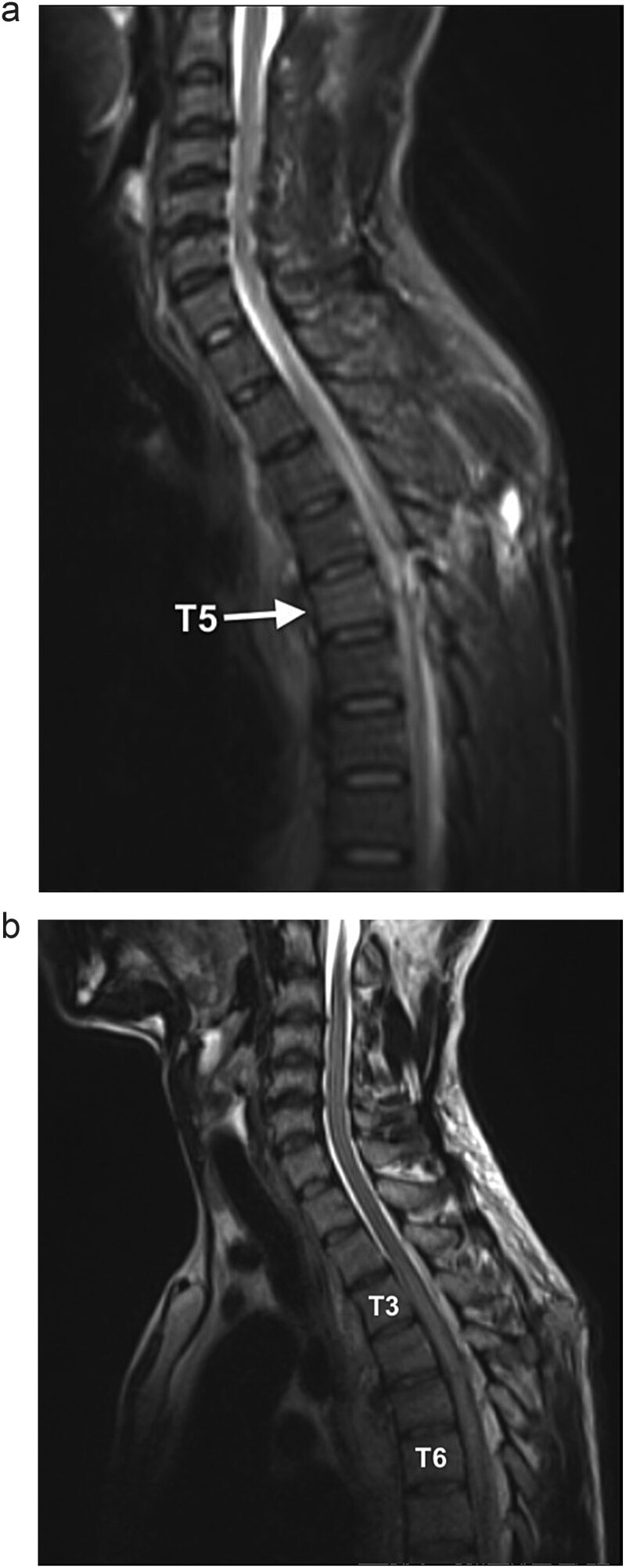
(a) Sagittal STIR MR shows a hyperintense signal along the dorsal mid-thoracic soft tissue wound tract, extending into the spinal canal with T5 level cord disruption and T5 vertebral body marrow oedema. (b) Sagittal T2WI MR demonstrates cord oedema as a hyperintense signal of the central cord (T3 to T6 level).

**Figure 2:**
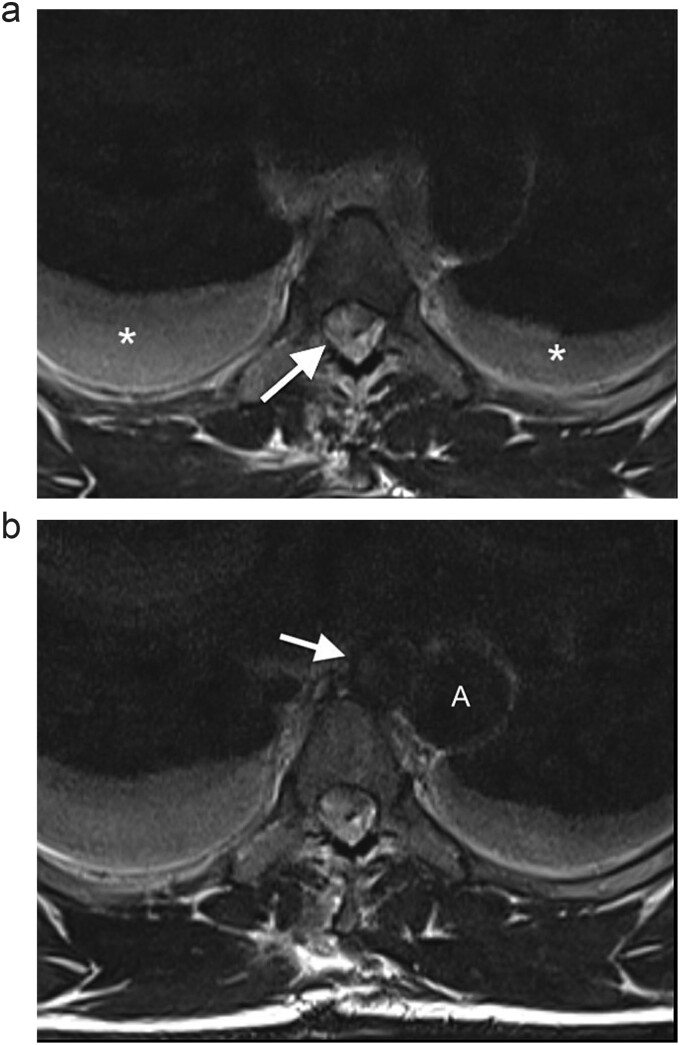
(a) Axial T2WI MR at the midthoracic level demonstrates an epidural hematoma in the right lateral spinal canal with compression of the cord (arrow) and bilateral hydrothoraces (*). (b) Axial T2WI MR shows a round lesion (arrow) inseparable from the medial aspect of the mid-thoracic aorta (A), with an isointense signal to the thoracic aorta.

On neurological examination, the patient was suspected of having Brown–Sequard syndrome, as power and light touch sensation was reduced in the right lower limb, with reduced sensation over the right lower abdomen. Furthermore, there was decreased pain sensation in the left lower limb. He had normal bladder function and normal sensation over the sacral area. The patient was reviewed by the Neurosurgical team and referred for an magnetic resonance imaging (MRI) of the spine. The MRI demonstrated a penetrating injury to the mid-thoracic spine with a right hemicord transection at the level of T5 (Fig. 1a). There was an associated right spinal epidural hematoma with resultant compression of the cord and cord oedema (Figs 1b and 2a). The Neurosurgery team elected to manage the injury conservatively.

**Figure 3:**
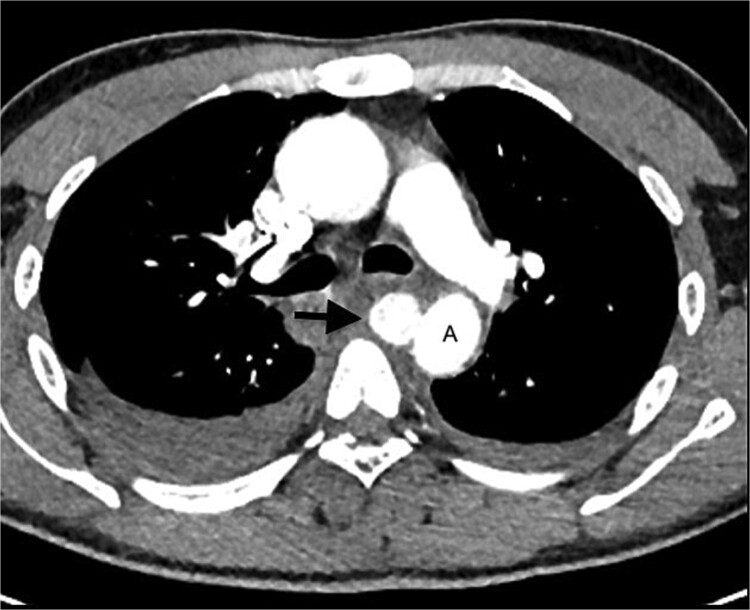
CT angiogram of the chest confirms a pseudoaneurysm (arrow) of the thoracic aorta (A), with surrounding mediastinal haematoma and bilateral haemothoraces.

The MRI also revealed a well-circumscribed lesion in the posterior mediastinum at the T4–5 level, inseparable from the medial aspect of the mid-thoracic aorta, at the level of the penetrating wound (Fig. 2b). These findings were concerning for a traumatic aortic injury, and the patient was therefore referred for an urgent computed tomography (CT) angiogram of the chest. The CT angiogram confirmed a pseudoaneurysm of the descending aorta, 3 mm in diameter, associated with a mediastinal hematoma and bilateral haemothoraces (Fig. 3). In retrospect, the aortic injury was an incidental finding. The vascular surgery team was consulted, and the patient was booked for an endovascular thoracic aorta repair on the following elective surgical list. The patient was admitted to our high care unit for monitoring while awaiting theatre. A labetalol infusion was initiated to control his blood pressure, and appropriate analgesia was administered. He remained haemodynamically normal during admission, and his haemoglobin level did not fall.

On performance of the thoracic endovascular aortic repair (TEVAR) procedure, a pseudoaneurysm was found in the descending aorta, 17 mm from the left subclavian artery. After endovascular access was obtained, a 30 × 26 mm tapered stent (length 108 mm) was deployed to the left of the left subclavian artery. The postoperative period was uneventful; the patient remained fully orientated and tolerated a full ward diet. He mobilized to a chair with the assistance of the physiotherapists, and rehabilitation was initiated for the monoplegia of his right lower limb. The monoplegia subsequently started to improve.

## Discussion

Stab wounds represent the most common mechanism of penetrating trauma in South Africa, with the thorax being the most commonly affected anatomical area [3]. In 85% of patients with injuries to the thoracic aorta, death occurs at the scene of injury. Of those patients who reach the hospital, up to 70% of them are unstable on arrival [1]. However, in low-velocity injuries (such as stab wounds), the injury is usually contained and may form a pseudoaneurysm [4]. In a patient with penetrating injury to the chest, clinical diagnosis of aortic injury is difficult, as signs and symptoms may be non-specific [5]. However, the treating doctor should have a high index of suspicion in penetrating injuries that cross the midline [4], as may occur in gunshot wounds. The diagnosis is made on imaging, with CT angiogram being the gold standard.

Endovascular repair is favoured over open repair in non-trauma patients with thoracic aortic aneurysms, because it carries a lower peri-operative mortality rate than open repair, as cross-clamping of the Aorta is avoided [6], and the rate of neurological morbidity from stroke and spinal cord ischaemia is also lower [1]. The complications associated with prolonged hospital stay are reduced in the TEVAR group, as these patients recover up to 50% faster in the postoperative period than those undergoing open surgery [1].

Guidelines from the Vascular Society of South Africa recommend TEVAR as first-line therapy for thoracic aorta injury secondary to blunt trauma [1]. In contrast, open surgery is considered the standard of care in penetrating Thoracic vascular injury, as most of these cases have haemodynamic instability and associated injuries that require surgical intervention [1]. A study published by the Journal of Vascular Surgery demonstrated that delayed endovascular aortic repair (more than 24 hours after injury) is associated with reduced mortality in patients with blunt thoracic aortic injury [4]. The patient in this case report, although a victim of penetrating thoracic aortic trauma presented 5 days after injury with a contained injury (aortic pseudoaneurysm) and was haemodynamically stable. Therefore, TEVAR was an option in this case.

Brown–Sequard syndrome is rare, with an incidence of < 5% of traumatic spinal cord injuries [7]. In South Africa, penetrating trauma accounts for 60% of all spinal cord injuries, with the knife being the most common assault weapon used in non-missile penetrating spinal cord injury [8]. Brown–Sequard syndrome has the best prognosis of all traumatic spinal cord injuries, with most patients regaining ambulatory function within a month [9]. The use of corticosteroids is controversial, and surgery is generally only indicated for patients with retained foreign bodies, cerebrospinal fluid leak or expanding lesions that require decompression [10]. Thus, management is focused on supportive care and early rehabilitation [10].

## Conclusion

This case report demonstrates an adult male with delayed presentation of Brown–Sequard Syndrome following a penetrating injury to the paraspinal area, with an incidental finding of injury to the descending aorta. The aortic injury was successfully managed with a minimally invasive procedure, and the morbidity associated with a thoracotomy was avoided. TEVAR can be successfully performed in penetrating trauma involving major vascular injuries.

## References

[1] Veller M (ed). Thoracic aortic interventions. In: Vascular Society of Southern Africa. Johannesburg: Vascular Society of Southern Africa; 2023. Available from: https://www.vascularsociety.co.za/wp-content/uploads/2024/03/Thoracic-aortic-interventions-2012.pdf

[2] Huang X, Chen F, Yu C, et al. A rare case of penetrating thoracic aortic injury. Int J Surg Case Rep 2023;106:108–84. 10.1016/j.ijscr.2023.108184 PMC10164878 37105024

[3] Bhana M, Fru P, Plani F. A long walk to freedom: the epidemiology of penetrating trauma in South Africa - analysis of 4 697 patients over a six-year period at Chris Hani Baragwanath academic hospital. S Afr J Surg 2022;60:77–83. 35851359

[4] Wall MJ, Tsai PI, Gilani R, et al. Open and endovascular approaches to aortic trauma. Tex Heart I J 2010;37:675–7. PMC3014142 21224943

[5] D'Souza D, Chieng R, Sharma R. et al. Thoracic Aortic Injury. Australia: Radiopaedia.org; 2023. 10.53347/rID-2171 (18 November 2023, date last accessed).

[6] Sarquis LM, Michaelis W, Santos Filho AL., et al. Endovascular approach to penetrating thoracic aortic injury – case report. J Vasc Bras 2020;19:e20200132. 10.1590/1677-5449.200132. 34211531 PMC8217996

[7] Shams S, Arain A. Brown-Sequard syndrome. In: StatPearls [Internet]. Florida: StatPearls Publishing; 2022. Available from: https://www.ncbi.nlm.nih.gov/books/NBK538135/ (21 December 2023, date last accessed). 30844162

[8] Kramer M, Acker A, Ohana N. Penetrating spinal cord injury. In: Essentials of Spinal Cord Injury Medicine. Germany: IntechOpen; 2018. Available from: https://www.intechopen.com/chapters/61364 (21 December 2023, date last accessed).

[9] Abdulqader M, Ismail M, Al-Khafaji A, et al. Brown-Sequard syndrome associated with a spinal cord injury caused by a retained screwdriver: a case report and literature review. Surg Neurol Int 2022;13:520. 10.25259/SNI_957_2022. 36447879 PMC9699854

[10] Rodríguez-Quintero J, Romero-Velez G, Pereira X, et al. Traumatic Brown-Séquard syndrome: modern reminder of a neurological injury – case report. BMJ 2020;13:236131. 10.1136/bcr-2020-236131. PMC7705369 33257359

